# Long-Term Improvement in a Chinese Cohort of Glucocorticoid-Resistant Childhood-Onset Myasthenia Gravis Patients Treated With Tacrolimus

**DOI:** 10.3389/fneur.2022.820205

**Published:** 2022-02-08

**Authors:** Zhuajin Bi, Yayun Cao, Jing Lin, Qing Zhang, Chenchen Liu, Mengcui Gui, Bitao Bu

**Affiliations:** ^1^Department of Neurology, Tongji Hospital, Tongji Medical College, Huazhong University of Science and Technology, Wuhan, China; ^2^Department of Radiology, Zhongnan Hospital of Wuhan University, Wuhan, China

**Keywords:** myasthenia gravis, children, tacrolimus, thymus type, pre-intervention status

## Abstract

**Objectives:**

To evaluate the long-term outcome of tacrolimus for childhood-onset myasthenia gravis (CMG) with an inadequate response to glucocorticoids, and investigate factors associated with favorable outcomes following tacrolimus treatment.

**Methods:**

A retrospective, observational cohort study was performed for CMG patients who had not improved satisfactorily after sufficient prednisone therapy for at least 8 weeks. All patients were given tacrolimus in doses of 2–3 mg for more than 6 months. The primary efficacy outcome was assessed using the prednisone dose, quantitative MG (QMG), and MG-activity of daily living (ADL) scores. The participants were divided into improved and unimproved groups based on changes in QMG scores to investigate the risk factors that affected tacrolimus efficacy.

**Results:**

A total of 149 glucocorticoid resistant CMG patients were finally enrolled in our study, with 113 (75.8%) responding well to tacrolimus (defined as minimal manifestation status or better). One month after initiating tacrolimus, there was a noticeable improvement in prednisone dose, QMG, and ADL scores, which continued to improve throughout the study. More importantly, the prednisone was eventually stopped in 89 of the patients (78.8%). Thymus type [odds ratio (OR) = 3.156, 95% confidence interval (CI) 1.427–6.978; *P* = 0.005] and pre-intervention status (OR = 0.284, 95%CI 0.109–0.741; *P* = 0.010) were independent predictors of tacrolimus efficacy after controlling for confounding factors in multiple logistic regression.

**Conclusion:**

The majority of glucocorticoid-resistant CMG patients have a good long-term prognosis after adding tacrolimus. Thymus type and pre-intervention status can serve as potential predictors affecting the efficacy of tacrolimus.

## Introduction

Myasthenia gravis (MG) is an acquired autoimmune disorder caused by antibodies that target the neuromuscular junction, leading to extraocular and/or systemic skeletal muscle weakness and fatigability (1, 2). The age distribution in MG seems different between Caucasians and East Asian populations (3, 4). In China, there are more than half of MG patients initially developed symptoms in childhood (5). The long-term treatment methods for MG patients usually include pyridostigmine, glucocorticoids (steroids) and immunosuppressants (IS) (6, 7). However, about 20–35% of the MG patients were insensitive to steroid therapy (8, 9). Furthermore, compared with adult-onset MG (AMG), childhood-onset myasthenia gravis (CMG) is more likely to develop resistance to steroids and suffer serious adverse drug reactions (ADRs) from long-term immunotherapy (5, 8). Alternative approaches with more satisfied efficacy and less serious ADRs are urgently needed for long-term use in CMG patients. Majority of CMG patients experienced fluctuating course characterized by remitting-relapsing pattern and slowly developed unresponsiveness to pyridostigmine and corticosteroids in China (8). The long-term outcome of CMG patients remained a major concern.

Tacrolimus, a kind of immunosuppressants by inhibiting interleukin-2 production, Th1 and Th17 responses, and T lymphocyte activation (10), had been suggested to satisfactorily and safely improve the symptoms of AMG patients who were unresponsive or intolerant to steroids (11, 12). However, clinical data about the efficacy and safety of tacrolimus in CMG is very limited, due to the difficulties with study design and recruitment of patients in sufficiently large numbers (5, 11, 13–15). In this study, we evaluated the efficacy and safety of tacrolimus in a cohort of steroid-resistant CMG patients. In addition, clinical predictors associated with favorable outcomes have been analyzed.

## Materials and Methods

### Study Design and Patient Selection

This study is a retrospective analysis of CMG patients from a single centre. Steroids resistant CMG patients were evaluated at Tongji hospital of Tongji Medical College, Huazhong University of Science and Technology from January 2015 to May 2020. The inclusion criteria were as follows: (1) patients with a confirmed diagnosis of MG based on the fatigable weakness of the skeletal muscles and at least one of the following positive results of the neostigmine test, repetitive nerve stimulation (RNS) test, or MG-related autoantibody test; (2) patients with onset age ≤ 14 years; (3) patients had an inadequate response to prednisone at doses of ≥0.75 mg/kg/day for at least 8 weeks prior to enrolment. The inadequate response was defined as meeting at least one of the following criteria (11, 16): ① QMG score or MG-ADL score improved by <25%; ② the steroids dosage failed to reduce; ③ the MGFA post-intervention state (PIS) didn't improve.

**Figure 1:**
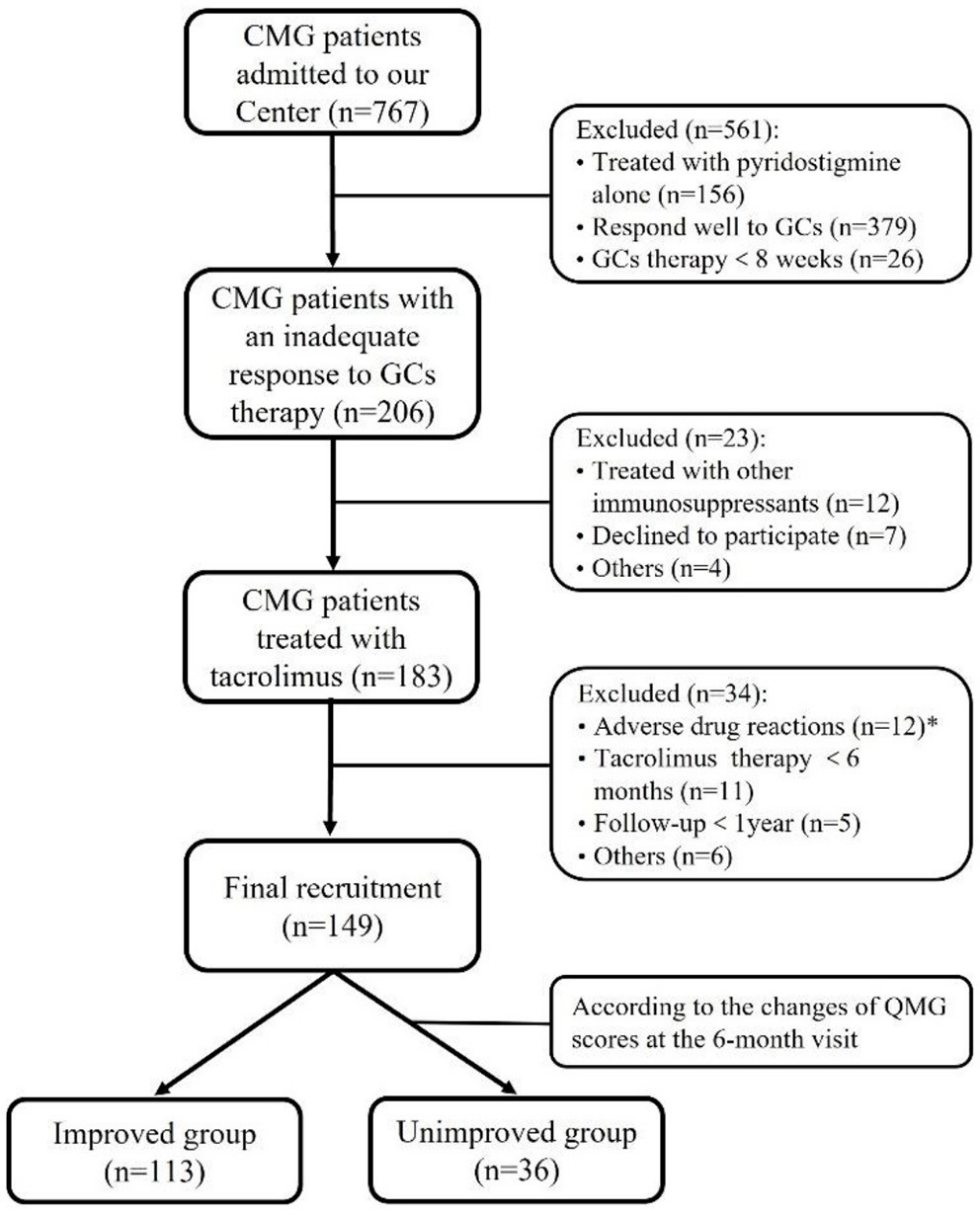
Flowchart of participants' recruitment. *During the follow-up throughout our study, 12 out of 183 (<7.5%) patients discontinued tacrolimus due to severe adverse drug reactions after a median of 2.8 months (ranged from 0.25 to 10.00 months): renal insufficiency in three, hepatic dysfunction in two, stomachache in two, tremor in two, hyperglycemia in one, infection in one, and allergic to tacrolimus in one.

Patients were excluded if they had any of the following conditions: (1) tacrolimus was not available because of complications, including diabetes, abnormal liver, and kidney function, or severe infectious diseases; (2) tacrolimus was withdrawn due to ADRs; (3) thymectomy or steroid-sparing agents were used within 3 months before the start of tacrolimus administration; (4) duration of follow-up is <1 year. In addition, to investigate factors that may potentially affect the efficacy of tacrolimus, the patients were divided into two groups based on the changes of QMG scores at the 6-month visit: the improved group with reduction of QMG score ≥25% and unimproved group with reduction of QMG score <25% (16, 17). Figure 1 depicted the selection procedure.

### Therapy, Evaluation, and Follow-Up

All participants were given 0.05 mg/kg/day of tacrolimus (Hangzhou Zhongmei Huadong Pharmaceutical Co., Ltd., H20094027), which was later adjusted to a trough level between 5 and 10 ng/dL according to the therapeutic effect (17). For patients with rapid tacrolimus metabolism, Wuzhi capsules, an ethanol herbal extract of Schisandra sphenanthera, were usually added to increased tacrolimus oral bioavailability (18). To investigate the long-term safety of tacrolimus, routine laboratory tests were performed every 4 weeks after tacrolimus administration to identify potential abnormalities in blood count, electrolytes, serum chemistry and blood glucose. The prednisone dose was gradually reduced by 5–10 mg per month after a noticeable improvement in symptoms. After a successful steroids withdrawal, the dose of tacrolimus was reduced by 0.5 mg every 3–6 months and subsequently removed after at least 6 months of MM or better status. If the clinical symptoms recurred, the dose of prednisone or tacrolimus was increased immediately until the symptoms improved and stabilized. Because prednisone was utilized to treat the majority of subjects (83.2%) in our study, their steroid dosages were expressed as equivalents to prednisone when oral steroids other than prednisone were used.

MG is classified as ocular MG (OMG) and generalized MG (GMG) according to symptoms within the first month of onset (19). The MGFA classification was used to evaluate the maximum clinical severity before tacrolimus administration, and MGFA-PIS was used to assess the clinical status at the last visit (20). In terms of the MGFA PIS, the category of “MM or better status” included minimal manifestation (MM) status, pharmacological remission (PR), and complete stable remission (CSR). Therapeutic effects were evaluated using the dose of prednisone, MG-ADL, and QMG scores. In addition, MG-ADL and QMG scores were performed at 3–4 h after the last dose of pyridostigmine to avoid the potential influence of cholinesterase inhibitors. Follow-up was conducted to evaluate the therapeutic effect for all patients and adjust the therapeutic agents was done once a month for the first 6 months of tacrolimus treatment and at least once every 3 months after that.

### Statistical Analysis

Numerical data are presented as mean ± standard deviation (SD) or median (interquartile range, IQR), and categorical data are presented as frequencies with absolute numbers and percentages. The changes in the titers of AChR-ab, prednisone dose, QMG, and ADL scores were accessed using Wilcoxon signed-rank test at each follow-up visit. Kaplan-Meier curve was used to visualize the discontinuation rate of steroids during the tacrolimus treatment. A univariate logistic regression analysis was applied to identify possible factors correlated with the efficacy of tacrolimus and entered variables with *p* values < 0.20 into the multivariate logistic regression analysis. Additionally, the spearman rank test was performed in all variables to reduce confounders if 2 variables have a correlation coefficient ≥0.5. After that, a multivariate logistic regression model was performed to determine predictors that independently affected the efficacy of tacrolimus, using a stepwise forward selection procedure with a 0.05 threshold for both inclusion and exclusion. All statistical analyses were performed with SPSS version 22.0 (SPSS Inc. Chicago, IL, USA), and two-tailed *P* < 0.05 was deemed to indicate statistical significance.

## Results

### Demographic Characteristics

**Figure 2:**
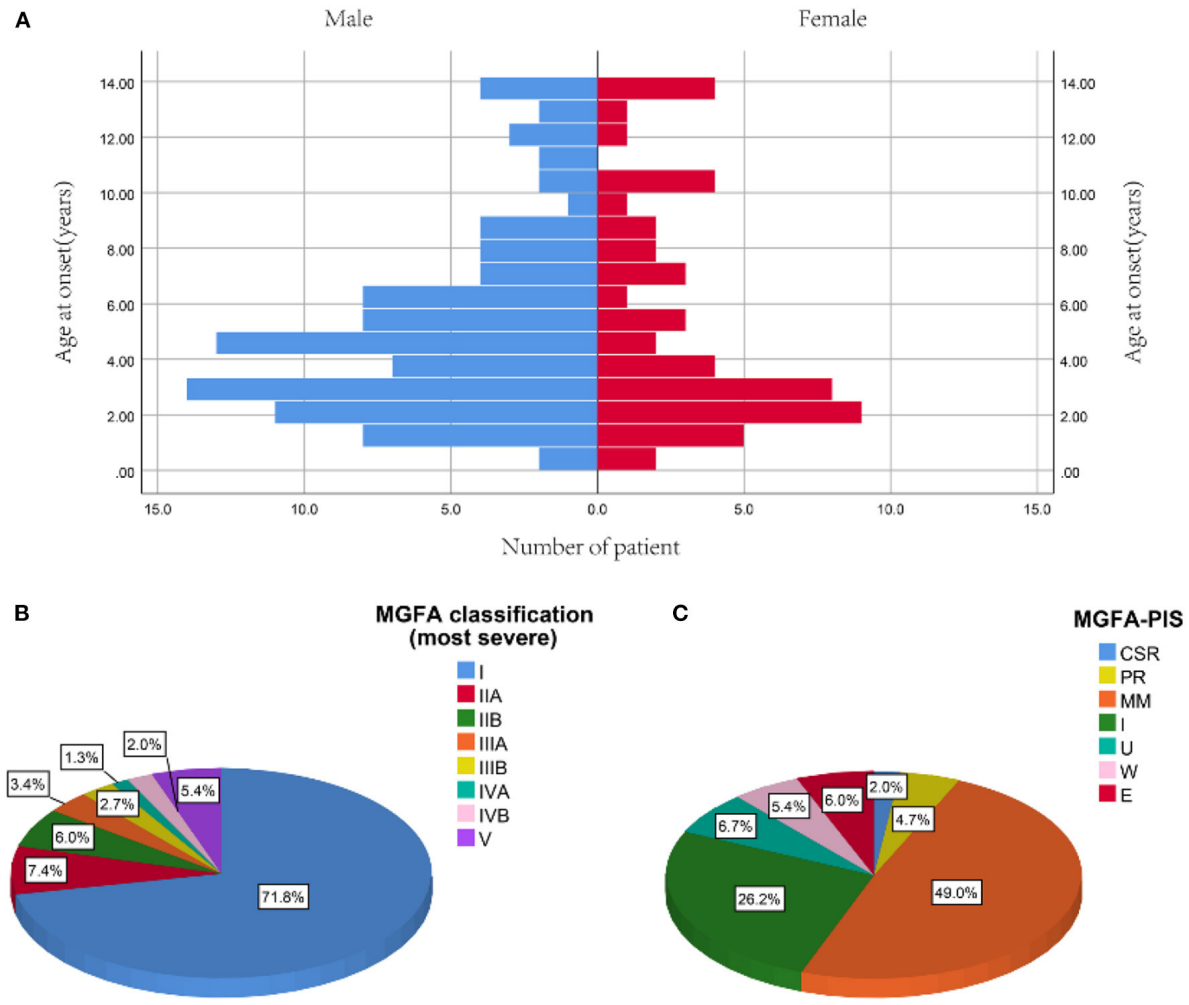
Profiles of study participants. **(A)** Distribution of onset age between male and female. **(B)** The most severe MGFA classification before tacrolimus administration. **(C)** MGFA-PIS on the last follow-up [median 12.9 years (IQR: 6.9, 19.2) from diagnosis]. Data are presented as the number or proportion of patients in each category. CSR, complete stable remission; E, exacerbation; MGFA, Myasthenia Gravis Foundation of America; MM, minimal manifestation; PIS, post-intervention state; PR, pharmacologic remission; U, unchanged; W, worse.

Among 767 CMG patients in our centre, 206 cases (35.2 %) were resistant to corticotherapy and 183 cases were then treated with tacrolimus. In addition, 12 out of 183 (<7.5%) patients discontinued tacrolimus due to severe ADRs after a median of 2.8 months (ranged from 0.25 to 10.00 months) (Figure 1). Thus, a total of 149 patients (median [IQR] age at onset: 4.4 [2.5, 7.4] years; 65.1% female) were enrolled in the study, with a follow-up for a median of 12.9 years (IQR: 6.9, 19.2) (Table 1). There was no significant difference in age of onset between males and females (*P* = 0.866) (Figure 2A). Of all patients, 140 patients (94.0%) showed only ocular symptoms at onset (MGFA class I). Ptosis was the most common initial presentation in 67.1% (100/149) of patients. 6.0% (9/149) of patients had generalized muscle weakness at onset. Besides, 33 out of the 140 OMG patients (20.4%) had transformed into GMG after a median of 12.0 years (IQR: 6.4–17.2). MG severity was classified as mild (MGFA I or II) in 127 patients (85.2%) and severe (MGFA III-V) in 22 patients (14.8%) before tacrolimus initiation (Figure 2B). The positive AChR-ab or MuSK-ab were detected in 113 (87.5%) patients and 1 (1.2%) patient, respectively. Thymus status was evaluated in all patients by chest computed tomography (CT) scan (2 thymomas, 39 thymus hyperplasia, 82 normal thymus) or thymus pathology (6 thymomas, 20 thymus hyperplasia). Thymectomy had been performed in 26 patients (17.4%) and the median (IQR) time from onset to thymectomy was 12.2 years (6.2, 15.7). One hundred and thirty two patients (79.0%) attained CSR, PR, MM or improvement at the last visit. However, 10 patients (6.7%) remained unchanged, 8 patients (5.4%) had clinically worsened symptoms, and 9 patients (6.0%) experienced exacerbated (Figure 2C).

**Table 1:** Baseline characteristics of 149 study participants.

**Characteristics**	**Patients**
**Gender**
Male	52 (34.9)
Female	97 (65.1)
Age at onset (years)	4.4 (2.5, 7.4)
≤ 5 years	84 (57.1)
5–10 years	42 (27.5)
> 10 years	23 (15.4)
Duration (years)	12.9 (7.4, 19.2)
Complicated with other AID	24 (16.1)
Neostigmine test (+)	144 (96.6)
RNS abnormalities	20/31
**Autoantibody status^a^**
AChR-ab (+)	113/149
MuSK-ab (+)	1/84
**Thymus type^b^**
Normal	82 (55.0)
Hyperplasia	59 (39.6)
Thymoma	9 (5.4)
Thymectomy	26 (17.4)
Age at thymectomy (years old)	16.0 (11.3, 20.6)
Time from onset to thymectomy (years)	12.2 (6.2, 15.7)
Ocular MG at onset	140 (94.0%)
Ptosis	100 (67.1)
Diplopia	11 (7.4)
Ptosis and diplopia	21 (14.1)
Ptosis and strabismus	8 (5.4)
Generalized MG at onset	9 (6.0%)
Limb weakness	3 (2.0)
Bulbar weakness	4 (2.7)
Limb and bullar weakness	2 (1.3)
Generalized disease development (years)^c^	12.0 (6.4, 17.2)
Within 2 years	3 (2.0)
After 2 years	30 (20.1)
Pre-intervention status
Unchanged	31 (20.8)
Worse	24 (16.1)
Exacerbation	94 (63.1)

*Data are given as n (%) or median (interquartile range)*.

a *The AChR-ab titers >0.50 nmol/L and MuSK-ab titers >0.05 nmol/L were defined as positive (RIA kit, RSR Limited, Cardiff, UK)*.

b *Thymus status was evaluated by chest computed tomography (CT) scan in non-thymectomized patients and thymus histology in thymectomized patients*.

c *Because only patients with ocular forms at onset can develop a generalized disease, the denominators are the number of patients with ocular forms at onset*.

*AChR-ab, anti-acetylcholine receptor antibodies; AID, autoimmune disease; MG, Myasthenia gravis; MGFA, Myasthenia Gravis Foundation of America; MuSK-ab, anti-muscle specific kinase autoantibody; RNS, repetitive nerve stimulation*.

### Efficacy Evaluation of Tacrolimus

All patients received a daily dose of 2–3 mg of tacrolimus with a mean trough concentration of 5.6 ± 1.5 ng/ml. The median age at the start of the tacrolimus was 15.2 years old (IQR, 9.3–22.2), and the median disease duration before initiating tacrolimus was 9.9 years (IQR, 4.1–16.3). After a mean follow-up of 3.16 ± 1.33 years, the tacrolimus dosage had been successfully tapered from 2.53 ± 0.74 to 1.55 ± 0.66 mg/day in 16 patients and withdrawn in 8 patients without any deterioration. The remaining 125 patients needed to maintain the initial tacrolimus dose to control the symptoms.

**Figure 3:**
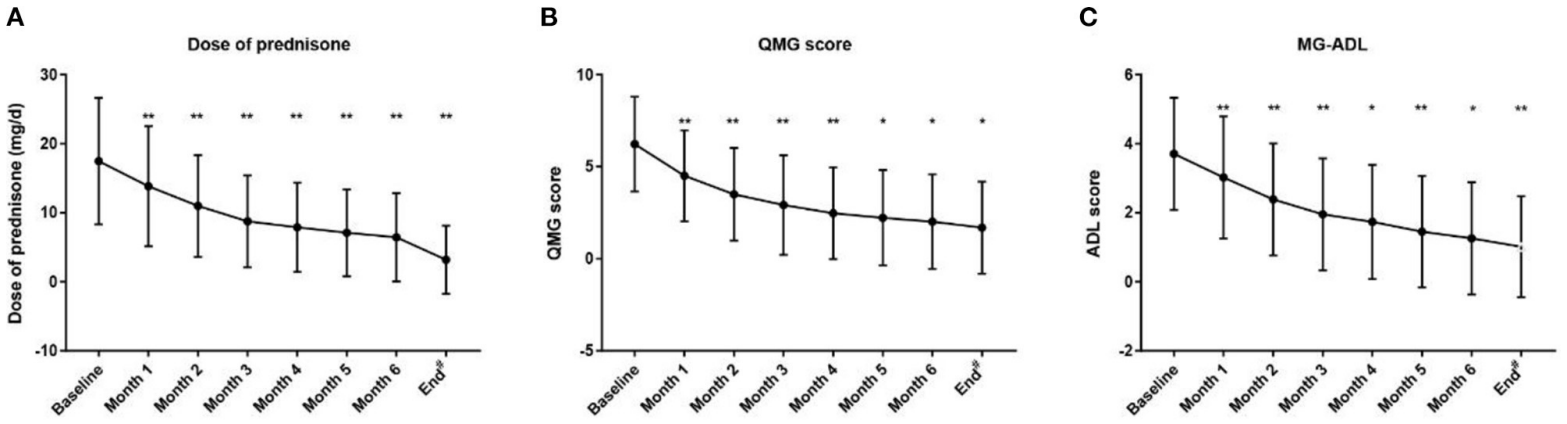
Changes of the prednisone dose, QMG score and MG-ADL score during treatment with tacrolimus. ^#^Mean follow-up of 3.16 ± 1.33 years (range 1.24–7.18 years). ADL, activity of daily living; QMG, quantitative MG. **(A)** Dose of prednisone, **(B)** QMG score, **(C)** MG-ADL score decreased gradually after initiation of tacrolimus treatment during the 6-month following-up (Dose of prednisone were 17.47 ± 9.16, 13.83 ± 8.69, 10.97 ± 7.38, 8.74 ± 6.65, 7.87 ± 6.46, 7.08 ± 6.28, 6.42 ± 6.39, 3.17 ± 4.93; QMG scores were 6.22 ± 2.58, 4.50 ± 2.47, 3.50 ± 2.52, 2.91 ± 2.71, 2.47 ± 2.49, 2.22 ± 2.59, 2.01 ± 2.57, 1.68 ± 2.49; and ADL scores were 3.70 ± 1.63, 3.02 ± 1.77, 2.38 ± 1.62, 1.95 ± 1.62, 1.73 ± 1.65, 1.45 ± 1.61, 1.26 ± 1.63, 1.01 ± 1.46 at the start of acrolimus treatment, 1, 2, 3, 4, 5, 6 month and the end of follow-up; respectively) (Compared with the last follow-up time point, *P < 0.05, **p < 0.001; 2-tailed Wilcoxon signed-rank test). *p < 0.05. **p < 0.01.

**Figure 4:**
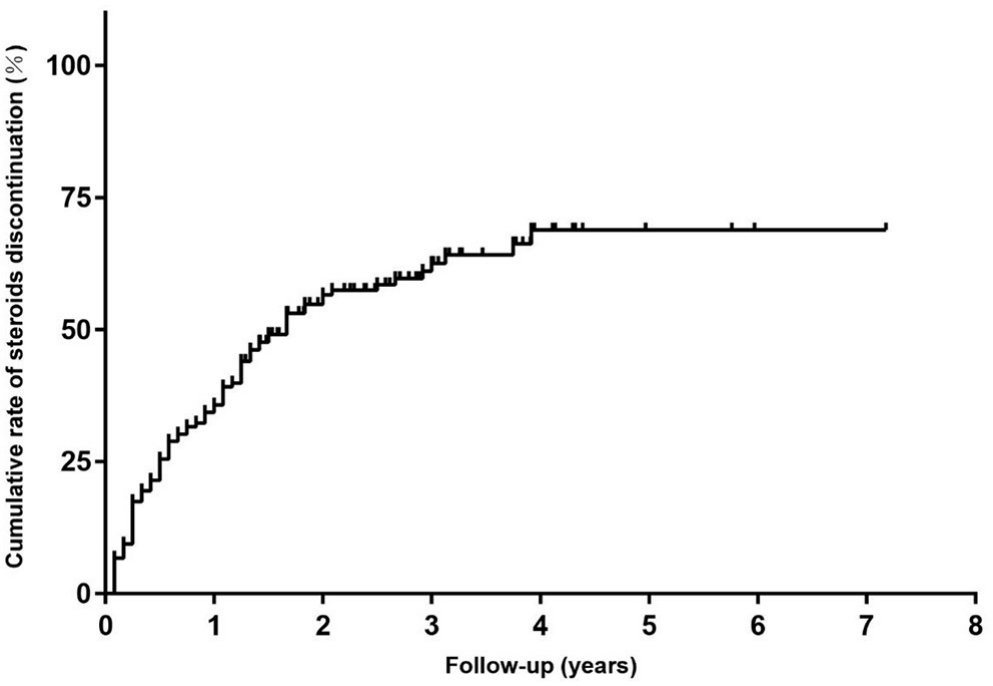
The Kaplan-Meier curve for time to discontinued steroids in children with steroid-resistant MG.

In addition, all patients had received prednisone for a median (IQR) duration of 2.0 (0.6, 4.5) years before tacrolimus and the median (IQR) age at prednisone initiation were 8.9 (4.5, 17.3) years old. The mean prednisone dosage significantly decreased after tacrolimus was added to treatment, from 17.47 ± 9.16 mg/day at baseline to 6.42 ± 6.39 mg/day at the 6-month visit (Figure 3A, *p* < 0.001). Furthermore, at 3, 6, 12, 24, and more than 24 months of follow-up, 26, 12, 15, 28, and 8 cases were withdrawn from prednisone due to improvement following tacrolimus treatment, respectively (Figure 4). Compared to the baseline, there was a statistically significant improvement in QMG and ADL scores at 1, 2, 3, 4, 5, and 6 months after initiating tacrolimus (Figures 3B,C, *p* < 0.05).

After an average of 0.9 years (range 0.5–1.8 years) of tacrolimus treatment, 41 of 113 (36.3%) anti-AChR antibody-positive cases were retested for anti-AChR antibodies, and the mean titers of AChR-ab were significantly decreased from 4.649 ± 2.564 to 2.283 ± 1.250 nmol/L (*P* < 0.001). However, no conversion from positive to negative for AChR-ab was observed in these patients. Moreover, only 1 of the 84 (1.2%) child with MG was positive for anti-MuSK antibody. This patient was a 5 year old girl who developed ptosis and diplopia followed by progressive limbs weakness, dysphagia, and dysarthria during the pyridostigmine and prednisone treatment. Thymic hyperplasia was identified on a chest CT scan. After 3 months of tacrolimus therapy, she was improved and no longer had any symptoms. This girl gradually stopped taking prednisone, and the MuSK-ab changed from positive to negative after one year.

### Factors That Might Influence the Efficacy of Tacrolimus in the Treatment of CMG

The patients were divided into two groups: improved MG group (*n* = 113) and unimproved MG group (*n* = 36). The common clinical features of the improved and unimproved patients were available in Supplementary Table 1. Gender, thymus type, thymectomy, the tacrolimus concentrations before tapering, and pre-intervention status were found to be associated with the clinical outcome using univariate logistic regression analysis. Although the thymectomy was found to be significant by univariate analysis, it was excluded from multivariate regression analysis due to its strong correlation with thymus type (Spearman correlation coefficient = 0.544, *P* < 0.001). Finally, multivariate logistic regression analysis indicated that thymus hyperplasia and pre-intervention status were independent predictors of tacrolimus efficacy. To be specific, thymic hyperplasia compromise therapeutic efficacy of tacrolimus compared with normal thymic (odds ratio [OR] = 3.140, 95% confidence interval [CI] =1.374–7.178; *P* = 0.007) but not thymoma (OR = 1.066, 95% CI = 0.113–10.085, *P* = 0.956). For pre-intervention status, CMG with exacerbated status had better therapeutic efficacy than those with unimproved status (OR = 0.284, 95% CI = 0.109–0.741, *P* = 0.010) (Table 2).

**Table 2:** Univariate and multivariate analysis for the influencing factors of the tacrolimus efficacy.

**Variable**	**Univariable**		**Multivariable**	
	**OR (95%CI)**	***P***	**OR (95%CI)**	***P***
Age at onset, y	1.011 (0.912, 1.120)	0.834		
Gender (male vs. female)	0.502 (0.233, 1.079)	0.078^*^		
Duration, y	0.502 (0.233, 1.079)	0.533		
Complicated with other AID	0.798 (0.275, 2.316)	0.678		
Neostigmine test (+)	1.011 (0.973, 1.051)	0.576		
Symptoms at onset^a^	1.621 (0.384, 6.841)	0.511		
**MGFA classification**
I II III IV V	1 [Reference] 1.482 (0.514, 4.273) (0.000, 0.000) 2.306 (0.364, 14.604) 3.458 (0.804, 14.868)	0.470 0.466 0.999 0.375 0.095		
QMG score before tacrolimus administration	1.000 (0.864, 1.158)	0.998		
ADL score before tacrolimus administration	1.051 (0.838, 1.318)	0.668		
AChR-ab titers, nmol/L	1.430 (0.566, 3.614)	0.449		
Thymus type				
Normal	1 [Reference]	0.012^**^	1 [Reference]	0.022^**^
Hyperplasia	3.156 (1.427, 6.978)	0.005	3.140 (1.374, 7.178)	0.007
Thymoma	0.758 (0.086, 6.691)	0.803	1.066 (0.113, 10.085)	0.956
Thymectomy	2.875 (1.176, 7.024)	0.021^*^		
Age at Pre administration, y	1.011(0.972, 1.051)	0.587		
Interval between onset and starting Pre, y	1.012 (0.969, 1.057)	0.590		
Duration of Pre before Tac, y	1.002 (0.903, 1.110)	0.976		
Age at Tac administration, y	1.012 (0.973, 1.052)	0.566		
Interval between onset and starting Tac, y	1.015 (0.971, 1.060)	0.514		
Tac concentrations before tapering, ng/mL	1.185 (0.920, 1.526)	0.189^*^		
Pre-intervention status				
Unimproved	1 [Reference]	0.004^**^	1 [Reference]	0.007^**^
Worse	1.131 (0.382, 3.353)	0.824	1.130 (0.364, 3.509)	0.832
Exacerbation	0.277 (0.111, 0.695)	0.006	0.284 (0.109, 0.741)	0.010

a *Patients were categorized as ocular MG (MGFA class I) and generalized MG (MGFA class II-V) according to the initial symptoms*.

*AChR-ab, acetylcholine receptor antibody; AID, autoimmune disease; MG, Myasthenia gravis; MGFA, Myasthenia Gravis Foundation of America; OR, odds ratio; Pre, prednisone; SD, standard deviation; Tac, tacrolimus*.

* *p < 0.20;*

** *p < 0.05*.

## Discussion

In this study, we reported the clinical data from CMG patients who did not achieve satisfactory therapeutic effects and then were treated with tacrolimus. The majority of subjects can respond well to tacrolimus, and nearly half attained MMS or better status at the end of follow-up (21). In contrast to previous studies, which found no gender bias in CMG patients in Asian populations (8, 22), our data showed a female preponderance, which was significantly lower than that the entire population of CMG patients treated at our center (seen in the Supplementary Table 2). This gender bias may be the result of a combination of sex hormones and genetic predisposition on the immunological function, and it represents disparities in therapeutic response to steroids between males and females (23). Similar to earlier studies that the majority of CMG patients only had ocular symptoms (8, 22, 24), the current study showed that 94.0% of recruited subjects had OMG. However, our cohorts had a higher rate of generalized conversion or severe MG (MGFA III-V) during pyridostigmine and steroids therapy. Therefore, alternative non-steroidal immune-suppressants with better efficacy is urgently needed to treat CMG patients with an inadequate response to steroids.

The QMG and MG-ADL scores were primarily used to assess the therapeutic efficacy of MG (20). Our findings revealed that these two markers began to improve 1 month after initialing tacrolimus and steadily improved throughout the study. This is in line with recent studies reporting that the therapeutic effect of tacrolimus can be shown within 4 weeks of commencing therapy, which is faster than other traditional IS, such as azathioprine, cyclophosphamide, and methotrexate (25–27). Another benefit of tacrolimus for patients with MG was that it allowed them to reduce their steroid dosages, however, there is a lot of individual variance (1, 28–30). Previous studies have demonstrated that the early favorable outcomes of tacrolimus may be caused by improving both the transport of steroids into the nucleus and the ability of steroid receptor to bind hormone (12, 29, 31). In this study, more than half patients were able to stop taking steroids after responding well to tacrolimus, while 21.2% patients needed a combination of low-doses steroids to keep their symptoms under control at the end of follow-up. Furthermore, previous studies have reported positive results with tacrolimus monotherapy, suggesting that tacrolimus can be used alone or in combination with steroid (21, 32).

AChR antibodies were found in the majority of the participants in our study, and the reduction of AChR-ab titers was accompanied by the clinical improvement after tacrolimus treatment, which was consistent with prior researches (12, 24, 29). By comparing the AChR-ab titers before enrollment between the improved and unimproved group, we were able to show that it was not an independent risk factor for tacrolimus efficacy. These findings suggest that, while AChR-ab levels do not correlate with tacrolimus efficacy, dynamic changes in AChR-ab titers are helpful to assess the symptom improvement and guide further treatment. In addition, MG patients with MuSK-ab (MuSK-MG) had a substantially greater probability of failure with traditional IS agents compared to patients with AChR-MG (10, 33). We effectively treated a severe generalized MuSK-ab-positive CMG patient with tacrolimus in our study, suggesting that tacrolimus might be a viable treatment for children with MuSK-MG (10).

Two clinical predictors of tacrolimus efficacy were identified statistically in our study: thymus type and pre-intervention status. Although the relationship between the thymus gland and MG is not yet fully understood (34). The thymus is thought to play an important role in the pathogenesis of MG. Our data showed that concomitant thymus hyperplasia was an independent risk factor for poor efficacy of tacrolimus in children with steroid-resistant MG, even in situation when thymectomy therapy had been used. One theory is that autoreactive T-lymphocytes exported from the aberrant germinal center in thymus hyperplasia might have remained in the periphery for a long period and then been activated to disrupt immunological homeostasis (35). However, it should be noted that thymus status was mostly assessed by CT or MRI scans in our study, which may have limited sensitivity for thymus hyperplasia and hence bias of the assessment of tacrolimus effectiveness (36). Furthermore, the pre-intervention state of the improved and unimproved group differed, indicating that patients with exacerbated status before enrollment were more likely to respond effectively to tacrolimus than those with unimproved status. In terms of MGFA pre-intervention state, the majority of exacerbated patients who had previously achieved MM or better status with steroids therapy, tended to develop acquired resistance to steroids; whereas the unimproved cases did not respond to steroids once steroid therapy was initiated. This might mean that patients with MG who had developed resistance to steroids had a greater response to tacrolimus than those who had an initially poor response to steroids. Additionally, a recent cohort study reported tacrolimus combined with steroids can improve clinical effectiveness and serve as medication maintenance to prevent disease relapses in MG patients (36). Finally, because many clinical factors did not correlate with tacrolimus efficacy in steroid-resistant CMG patients, future researches should focus on biochemical and immunological indicators like NF-κB transcriptional activity, FK506-binding proteins (FKBPs), and abnormal T cell selection and activation (30, 33).

Even when the symptoms had been adequately controlled, most patients in our study were hesitant to stop taking tacrolimus because of the protracted illness course and significant chance of relapse (37). Tacrolimus dosage was successfully tapered in 24 (21.2%) of the 113 well-controlled patients in our cohorts without exacerbating their condition. In contrast to other studies which reported a higher incidence of tacrolimus-related ADRs, ranging from 42.5–87.5% (1, 11, 28, 38). Our data showed that <7.5% patients had ADRs after an average follow-up of 3 years, which may be related to the use of lower doses of tacrolimus. All the ADRs occurred within 10 months of tacrolimus treatment and were resolved when tacrolimus was discontinued. Therefore, we conclude that long-term tacrolimus usage in children with steroid-resistant MG is relatively safe.

In conclusion, children with steroid-resistant MG displayed distinct clinical characteristics. Although tacrolimus improved symptoms in the majority of steroid-resistant CMG patients with few adverse effects, some patients still did not react well to tacrolimus. Clinically independent factors affecting tacrolimus efficacy include thymus hyperplasia and pre-intervention status. And we are currently working on a follow-up study to explore the underlying immunological mechanism of therapeutic failure in patients who haven't responded to steroids or tacrolimus.

## Data Availability Statement

The raw data supporting the conclusions of this article will be made available by the authors, without undue reservation.

## Ethics Statement

The studies involving human participants were reviewed and approved by the Committee of Clinical Investigation at Tongji Hospital, Tongji Medical College, Huazhong University of Science and Technology, Wuhan China (NO. TJ-IRB20190414). Written informed consent to participate in this study was provided by the participants' legal guardian/next of kin.

## Author Contributions

ZB: acquired the data and drafted the manuscript. YC, JL, and QZ: interpreted the data and made suggestions for improvement. MG and BB: designed the study and revised the manuscript. All authors contributed to the article and approved the submitted version.

## Funding

This study was supported by the National Natural Science Foundation of China (Grant No: 8187051428).

## Conflict of Interest

The authors declare that the research was conducted in the absence of any commercial or financial relationships that could be construed as a potential conflict of interest.

## Publisher's Note

All claims expressed in this article are solely those of the authors and do not necessarily represent those of their affiliated organizations, or those of the publisher, the editors and the reviewers. Any product that may be evaluated in this article, or claim that may be made by its manufacturer, is not guaranteed or endorsed by the publisher.

## References

[1] Sanders DB Wolfe GI Benatar M Evoli A Gilhus NE Illa I. International consensus guidance for management of myasthenia gravis: executive summary. Neurology. (2016) 87:419–25. 10.1212/WNL.0000000000002790 27358333 PMC4977114

[2] Dalakas MC. Immunotherapy in myasthenia gravis in the era of biologics. Nat Rev Neurol. (2019) 15:113–24. 10.1038/s41582-018-0110-z 30573759

[3] He D Zhang H Xiao J Zhang X Xie M Pan D. Molecular and clinical relationship between live-attenuated Japanese encephalitis vaccination and childhood onset myasthenia gravis. Ann Neurol. (2018) 84:386–400. 10.1002/ana.25267 30246904 PMC6175482

[4] Zhang X Yang M Xu J Zhang M Lang B Wang W. Clinical and serological study of myasthenia gravis in HuBei Province, China. J Neurol Neurosurg Psychiatry. (2007) 78:386–90. 10.1136/jnnp.2006.100545 17088330 PMC2077769

[5] Liu C Gui M Cao Y Lin J Li Y Ji S. Tacrolimus improves symptoms of children with myasthenia gravis refractory to prednisone. Pediatr Neurol. (2017) 77:42–47. 10.1016/j.pediatrneurol.2017.08.014 29074055

[6] Yoshikawa H Kiuchi T Saida T Takamori M. Randomised, double-blind, placebo-controlled study of tacrolimus in myasthenia gravis. J Neurol Neurosurg Psychiatry. (2011) 82:970–7. 10.1136/jnnp-2011-300148 21784757

[7] Morren J Li Y. Maintenance immunosuppression in myasthenia gravis, an update. J Neurol Sci. (2020) 410:116648. 10.1016/j.jns.2019.116648 31901719

[8] Gui M Luo X Lin J Li Y Zhang M Zhang X. Long-term outcome of 424 childhood-onset myasthenia gravis patients. J Neurol. (2015) 262:823–30. 10.1007/s00415-015-7638-2 25588729

[9] Imai T Suzuki S Nagane Y Uzawa A Murai H Utsugisawa K. Reappraisal of oral steroid therapy for Myasthenia Gravis. Front Neurol. (2020) 11:868. 10.3389/fneur.2020.00868 32982912 PMC7477376

[10] Li Y Guptill JT Russo MA Massey JM Juel VC Hobson-Webb LD. Tacrolimus inhibits Th1 and Th17 responses in MuSK-antibody positive myasthenia gravis patients. Exp Neurol. (2019) 312:43–50. 10.1016/j.expneurol.2018.11.006 30472069 PMC6390960

[11] Zhou L Liu W Li W Li H Zhang X Shang H. Tacrolimus in the treatment of myasthenia gravis in patients with an inadequate response to glucocorticoid therapy: randomized, double-blind, placebo-controlled study conducted in China. Ther Adv Neurol Disord. (2017) 10:315–25. 10.1177/1756285617721092 28861121 PMC5557184

[12] Shimojima Y Matsuda M Gono T Ishii W Tokuda T Ikeda S. Tacrolimus in refractory patients with myasthenia gravis: coadministration and tapering of oral prednisolone. J Clin Neurosci. (2006) 13:39–44. 10.1016/j.jocn.2004.12.008 16307880

[13] Ning YM Sánchez ER. Potentiation of glucocorticoid receptor-mediated gene expression by the immunophilin ligands FK506 and rapamycin. J Biol Chem. (1993) 268:6073–6. 10.1016/S0021-9258(18)53220-8 7681058

[14] Mori T Mori K Suzue M Ito H Kagami S. Effective treatment of a 13-year-old boy with steroid-dependent ocular myasthenia gravis using tacrolimus. Brain Dev. (2013) 35:445–8. 10.1016/j.braindev.2012.06.012 22840813

[15] Kakisaka Y Haginoya K Yokoyama H Ishitobi M Wakusawa K Sato I. Successful treatment of a 2-year-old girl with intractable myasthenia gravis using tacrolimus. Brain Dev. (2006) 28:534–6. 10.1016/j.braindev.2006.02.001 16564661

[16] Liu GC Gao BL Yang HQ Qi GY Liu P. The clinical absolute and relative scoring system-a quantitative scale measuring myasthenia gravis severity and outcome used in the traditional Chinese medicine. Complement Ther Med. (2014) 22:877–86. 10.1016/j.ctim.2014.08.003 25440379

[17] Cruz JL Wolff ML Vanderman AJ Brown JN. The emerging role of tacrolimus in myasthenia gravis. Ther Adv Neurol Disord. (2015) 8:92–103. 10.1177/1756285615571873 25922621 PMC4356660

[18] Zhang H Bu F Li L Jiao Z Ma G Cai W. Prediction of drug-drug interaction between tacrolimus and principal ingredients of wuzhi capsule in chinese healthy volunteers using physiologically-based pharmacokinetic modelling. Basic Clin Pharmacol Toxicol. (2018) 122:331–40. 10.1111/bcpt.12914 28945011

[19] Ding J Zhao S Ren K Dang D Li H Wu F. Prediction of generalization of ocular myasthenia gravis under immunosuppressive therapy in Northwest China. BMC Neurol. (2020) 20:238. 10.1186/s12883-020-01805-1 32527235 PMC7288410

[20] Jaretzki A III Barohn RJ Ernstoff RM Kaminski HJ Keesey JC Penn AS. Myasthenia gravis: recommendations for clinical research standards. Task Force of the Medical Scientific Advisory Board of the Myasthenia Gravis Foundation of America. Neurology. (2000) 55:16–23. 10.1212/WNL.55.1.16 10891897

[21] Yagi Y Sanjo N Yokota T Mizusawa H. Tacrolimus monotherapy: a promising option for ocular myasthenia gravis. Eur Neurol. (2013) 69:344–5. 10.1159/000347068 23549260

[22] Huang X Li Y Feng H Chen P Liu W. Clinical characteristics of juvenile myasthenia gravis in Southern China. Front Neurol. (2018) 9:77. 10.3389/fneur.2018.00077 29535672 PMC5835068

[23] Evoli A. Acquired myasthenia gravis in childhood. Curr Opin Neurol. (2010) 23:536–40. 10.1097/WCO.0b013e32833c32af 20581680

[24] Hong Y Skeie GO Zisimopoulou P Karagiorgou K Tzartos SJ Gao X. Juvenile-onset myasthenia gravis: autoantibody status, clinical characteristics and genetic polymorphisms. J Neurol. (2017) 264:955–62. 10.1007/s00415-017-8478-z 28364296

[25] Tindall RS Phillips JT Rollins JA Wells L Hall K. A clinical therapeutic trial of cyclosporine in myasthenia gravis. Ann N Y Acad Sci. (1993) 681:539–51. 10.1111/j.1749-6632.1993.tb22937.x 8357194

[26] Pasnoor M He J Herbelin L Burns TM Nations S Bril V. A randomized controlled trial of methotrexate for patients with generalized myasthenia gravis. Neurology. (2016) 87:57–64. 10.1212/WNL.0000000000002795 27306628 PMC4932232

[27] Palace J Newsom-Davis J Lecky B. A randomized double-blind trial of prednisolone alone or with azathioprine in myasthenia gravis. Myasthenia Gravis Study Group. Neurology. (1998) 50:1778–83. 10.1212/WNL.50.6.1778 9633727

[28] Zhao CB Zhang X Zhang H Hu XQ Lu JH Lu CZ. Clinical efficacy and immunological impact of tacrolimus in Chinese patients with generalized myasthenia gravis. Int Immunopharmacol. (2011) 11:519–24. 10.1016/j.intimp.2010.12.012 21195813

[29] Ponseti JM Gamez J Azem J López-Cano M Vilallonga R Armengol M. Tacrolimus for myasthenia gravis: a clinical study of 212 patients. Ann N Y Acad Sci. (2008) 1132:254–63. 10.1196/annals.1405.000 18096852

[30] Erlejman AG De Leo SA Mazaira GI Molinari AM Camisay MF Fontana V. NF-κB transcriptional activity is modulated by FK506-binding proteins FKBP51 and FKBP52: a role for peptidyl-prolyl isomerase activity. J Biol Chem. (2014) 289:26263–76. 10.1074/jbc.M114.582882 25104352 PMC4176250

[31] Li JW Fang F Ren XT Zhang WH Yang XY Ren CH. Clinical effect of tacrolimus in the treatment of myasthenia gravis in children. Zhongguo Dang Dai Er Ke Za Zhi. (2020) 22:964–9. 10.7499/j.issn.1008-8830.2004215 32933627 PMC7499454

[32] Fan Z Li Z Shen F Zhang X Lei L Su S. Favorable effects of tacrolimus monotherapy on myasthenia gravis patients. Front Neurol. (2020) 11:594152. 10.3389/fneur.2020.594152 33193063 PMC7652845

[33] Gilhus NE Skeie GO Romi F Lazaridis K Zisimopoulou P Tzartos S. Myasthenia gravis - autoantibody characteristics and their implications for therapy. Nat Rev Neurol. (2016) 12:259–68. 10.1038/nrneurol.2016.44 27103470

[34] Marx A Pfister F Schalke B Saruhan-Direskeneli G Melms A Ströbel P. The different roles of the thymus in the pathogenesis of the various myasthenia gravis subtypes. Autoimmun Rev. (2013) 12:875–84. 10.1016/j.autrev.2013.03.007 23535159

[35] Fujii Y. Thymus, thymoma and myasthenia gravis. Surg Today. (2013) 43:461–6. 10.1007/s00595-012-0318-2 22948665

[36] Zhang C Bu B Yang H Wang L Liu W Duan RS. Immunotherapy choice and maintenance for generalized myasthenia gravis in China. CNS Neurosci Ther. (2020) 26:1241–54. 10.1111/cns.13468 33103369 PMC7702233

[37] Nishida Y Takahashi YK Kanai T Nose Y Ishibashi S Sanjo N. Safety of tapering tacrolimus dose in patients with well-controlled anti-acetylcholine receptor antibody-positive myasthenia gravis. Eur J Neurol. (2020) 27:100–4. 10.1111/ene.14039 31309642

[38] Kim YH Shin HY Kim SM. Long-term safety and efficacy of tacrolimus in myasthenia gravis. Yonsei Med J. (2019) 60:633–9. 10.3349/ymj.2019.60.7.633 31250577 PMC6597475

