# Supplementary material for: Long-Term Improvement in a Chinese Cohort of Glucocorticoid-Resistant Childhood-Onset Myasthenia Gravis Patients Treated With Tacrolimus
Source: Front Neurol. 2022 Feb 8;13:820205. doi: 10.3389/fneur.2022.820205 (PMC8860838; doi:10.3389/fneur.2022.820205)
Supplement: Supplementary file 1 [file Table_1.DOCX]

**Table S1**: Comparison of clinical data between improved group and unimproved group of tacrolimus therapy.

| Variable | Total  (n=149) | Improved  (n=113) | Unimproved  (n=36) | *P* value |
| --- | --- | --- | --- | --- |
| Age at onset, y | 4.43 (2.52, 7.37) | 4.47 (2.66, 7.06) | 4.14 (2.13, 8.23) | 0.851 |
| Sex (female/male) | 97/52 | 78/35 | 19/17 | 0.075 |
| Duration, y | 12.92 (7.42, 19.19) | 11.89 (6.71, 19.19) | 15.98 (8.92, 19.42) | 0.166 |
| Complicated with other AID | 24 (16.1) | 19 (16.8) | 5 (13.9) | 0.678 |
| Neostigmine test (+) | 144 (96.6) | 110 (97.3) | 34 (94.4) | 0.595 |
| AChR-ab titers | 2.844±3.237 | 3.790±4.472 | 2.542±2.686 | 0.197 |
| Thymus type  Normal  Hyperplasia  Thymoma | 82 (55.0)  59 (39.6)  8 (5.4) | 69 (61.1)  37 (32.7)  7 (6.2) | 13 (36.1)  22 (61.1)  1 (2.8) | 0.011* |
| Thymusectomy | 26 (17.4) | 15 (13.3) | 11 (30.6) | 0.017* |
| Symptoms at onset  Ptosis  Diplopia  Ptosis and diplopia  Ptosis and strabismus  Limb weakness  Bulbar weakness  Limb and bullar weakness | 100 (67.1)  11 (7.4)  21 (14.1)  8 (5.4)  3 (2.0)  4 (2.7)  2 (1.3) | 82 (72.5)  9 (8.0)  13 (11.5)  3 (2.6)  2 (1.8)  2 (1.8)  2 (1.8) | 18 (50.0)  2 (5.5)  8 (22.2)  5 (13.9)  1 (2.8)  2 (5.6)  0 (0.0) | 0.058 |
| MGFA classification before Tac therapy  I  II  III  IV  V | 107 (71.8)  20 (13.4)  9 (6.0)  5 (3.4)  8 (5.4) | 83 (73.4)  14 (12.4)  9 (8.0)  3 (2.7)  4 (3.5) | 24 (66.7)  6 (16.7)  0 (0.0)  2 (5.5)  4 (11.1) | 0.196 |
| QMG score before tacrolimus administration | 6.22±2.58 | 6.22±2.74 | 6.22±1.99 | 0.998 |
| ADL score before tacrolimus administration | 3.70±1.63 | 3.67±1.73 | 3.81±1.26 | 0.618 |
| Interval between onset and starting Pre, y | 2.03 (0.06, 11.84) | 1.46 (0.00, 11.38) | 4.85 (0.88, 12.53) | 0.072 |
| Age at Pre administration, y | 8.98 (4.54, 17.31) | 8.26 (4.54, 15.43) | 9.65 (3.71, 21.67) | 0.106 |
| Duration of Pre before Tac, y | 2.00 (0.64, 4.50) | 2.00 (0.64, 4.60) | 1.90 (0.55, 4.45) | 0.708 |
| Age at Tac administration, y | 15.21 (9.33, 22.20) | 14.59 (9.07, 20.67) | 17.66 (9.72, 23.55) | 0.292 |
| Interval between onset and starting Tac, y | 9.93 (4.12, 16.32) | 9.15 (3.39, 15.50) | 12.75 (5.98, 16.92) | 0.155 |
| Tac concentrations before tapering, ng/mL, mean±SD | 5.64±1.49 | 5.55±1.52 | 5.95±1.37 | 0.188 |
| Pre-intervention status  Unchanged  Worse  Exacerbation | 31 (20.8)  24 (16.1)  94 (63.1) | 19  14  80 | 12  10  14 | 0.002* |

Note

Data are given as n (%) or median (IQR).

Analysis of continuous data was done with the Mann–Whitney U test or Student t test. Analysis of dichotomous data was done with the χ2 test or Fisher's exact test as appropriate. * : *p*< 0.05.

Abbreviations: AChR-ab, anti-acetylcholine receptor antibodies; AID, autoimmune disease; MG, Myasthenia gravis; MGFA, Myasthenia Gravis Foundation of America; Pre, prednisone; SD, standard deviation; Tac, tacrolimus.

**Table S2**: Comparison of clinical characteristics between enrolled participants and overall CMG patients.

| Characteristics | Whole group  (n=767) | Tacrolimus group  (n=149) | *P* value |
| --- | --- | --- | --- |
| Gender  Male  Female | 342 (44.6)  425 (55.4) | 52 (34.9)  97 (65.1) | 0.029* |
| Age at onset (years)  ≤ 5 years  5–10 years    > 10 years | 472 (61.5)  200 (26.1)  95 (12.4) | 84 (56.4)  42 (39.4)  23 (19.2) | 0.238  0.593  0.309 |
| Duration (years) | 10.7 (7.2, 17.7) | 12.9 (7.4, 19.2) | 0.126 |
| Complicated with other AID | 84 (9.2) | 24 (16.1) | 0.074 |
| Neostigmine test (+) | 749 (97.7) | 144 (96.6) | 0.471 |
| RNS abnormalities | 55/133 | 20/31 | 0.020* |
| Autoantibody status^a^  AChR-ab (+)  MuSK-ab (+) | 296/419  2/228 | 113/149  1/84 | 0.225  0.801 |
| Thymus type^b^  Normal  Hyperplasia  Thymoma | 594 (77.4)  152 (19.8)  21 (2.7) | 97 (58.1)  61 (36.5)  9 (5.4) | 0.001* *  ＜0.001* *  0.046* |
| Thymectomy | 61 (8.0) | 26 (17.4) | ＜0.001* * |
| Age at thymectomy (years old) | 16.0 (12.5, 19.8) | 16.0 (11.3, 20.6) | 0.760 |
| Time from onset to thymectomy (years) | 10.0 (3.0, 15.3) | 12.2 (6.2, 15.7) | 0.335 |
| MGFA classification at onset  Ocular MG  Generalized MG | 752 (98.0)  15 (2.0) | 140 (94.0)  9(6.0) | 0.004* * |
| Generalized disease development^c^ | 108 (14.4) | 33 (23.6) | 0.006* * |
| Time from Ocular MG to Generalized MG (years) | 9.5 (5.0, 17.0) | 12.0 (6.4, 17.2) | 0.254 |
| MGFA classification (most severe)  I  II  III  IV  V | 643 (83.8)  76 (8.3)  19 (2.5)  12 (1.6)  17 (2.2) | 107 (71.8)  20 (13.4)  9 (6.0)  5 (3.4)  8 (5.4) | ＜0.001* *  0.200  0.021*  0.138  0.031* |
| Post-intervention status  MM or better  Improved  Unchanged  Worse  Exacerbation | 574 (74.8)  72 (9.4)  42 (5.5)  54 (7.0)  25 (3.3) | 83 (55.7)  39 (26.2)  10 (6.7)  8 (5.4)  9 (6.0) | ＜0.001* *  ＜0.001* *  0.551  0.457  0.100 |

Note

Data are given as n (%) or median (IQR).

Analysis of continuous data was done with the Mann–Whitney U test. Analysis of dichotomous data was done with the χ2 test or Fisher's exact test as appropriate. * : *p*< 0.05; **:*p* < 0.01.

^a^ The AChR-ab titers >0.50 nmol/L and MuSK-ab titers >0.05 nmol/L was considered to be positive.

^b^ Thymus status was evaluated by chest computed tomography (CT) or thymus pathology in patients undergoing thymectomy.

^c^ Because only patients with ocular forms at onset can develop a generalized disease, the denominators are the number of patients with ocular forms at onset.

Abbreviations: AChR-ab, anti-acetylcholine receptor antibodies; AID, autoimmune disease; CSR, complete stable remission; MG, Myasthenia gravis; MGFA, Myasthenia Gravis Foundation of America; MM, minimal manifestation; MuSK-ab, anti-muscle specific kinase autoantibody; PR, pharmacologic remission; RNS, repetitive nerve stimulation.
